# Dynamic Reweighting of Three Modalities for Sensor Fusion

**DOI:** 10.1371/journal.pone.0088132

**Published:** 2014-01-31

**Authors:** Sungjae Hwang, Peter Agada, Tim Kiemel, John J. Jeka

**Affiliations:** 1 Department of Kinesiology, Temple University, Philadelphia, Pennsylvania, United States of America; 2 Bioengineering, Temple University, Philadelphia, Pennsylvania, United States of America; 3 Department of Kinesiology, University of Maryland, College Park, Maryland, United States of America; McGill University, Canada

## Abstract

We simultaneously perturbed visual, vestibular and proprioceptive modalities to understand how sensory feedback is re-weighted so that overall feedback remains suited to stabilizing upright stance. Ten healthy young subjects received an 80 Hz vibratory stimulus to their bilateral Achilles tendons (stimulus turns on-off at 0.28 Hz), a ±1 mA binaural monopolar galvanic vestibular stimulus at 0.36 Hz, and a visual stimulus at 0.2 Hz during standing. The visual stimulus was presented at different amplitudes (0.2, 0.8 deg rotation about ankle axis) to measure: the change in gain (weighting) to vision, an intramodal effect; and a change in gain to vibration and galvanic vestibular stimulation, both intermodal effects. The results showed a clear intramodal visual effect, indicating a de-emphasis on vision when the amplitude of visual stimulus increased. At the same time, an intermodal visual-proprioceptive reweighting effect was observed with the addition of vibration, which is thought to change proprioceptive inputs at the ankles, forcing the nervous system to rely more on vision and vestibular modalities. Similar intermodal effects for visual-vestibular reweighting were observed, suggesting that vestibular information is not a “fixed” reference, but is dynamically adjusted in the sensor fusion process. This is the first time, to our knowledge, that the interplay between the three primary modalities for postural control has been clearly delineated, illustrating a central process that fuses these modalities for accurate estimates of self-motion.

## Introduction

Control of human upright stance during standing requires sensory input from multiple sources to detect center of gravity of gravity excursions and to generate appropriate muscle responses for upright stance control. Without appropriate knowledge of self-orientation, equilibrium control is severely compromised [1]. Estimation of body position/velocity (i.e., self-motion) is heavily dependent upon the integration of information from multiple sensory modalities including visual, vestibular and somatosensory (touch, pressure, proprioception); as evidenced by numerous studies that have shown that the stimulation of visual [2]–[7], proprioceptive [8]–[11], or vestibular systems [12]–[15] evoke body sway.

Numerous studies have demonstrated that the integration of sensory information (i.e., sensor fusion) appears to be dynamically regulated to adapt to changing environmental conditions and the available sensory information, a process referred to as “sensory reweighting” [1], [16]–[23]. Sensory reweighting is the process through which the nervous system changes the “emphasis” of a particular sensory input due to neurological injury or when environmental conditions change. For example, during eyes-closed stance on a fixed, level surface, the primary sensory source for information about body orientation in space is proprioceptive, but under conditions where the platform moves, the primary source of sensory information shifts from proprioceptive to vestibular [16], [23]. Most of these previous studies stimulated an individual sensory system or the combined influence of two sensory systems. Here we investigate the combined influence of all three sensory systems for the control of human upright posture. This is the first time, to our knowledge, that the interplay between the three primary modalities for postural control has been clearly delineated, illustrating a central process that fuses these modalities for accurate estimates of self-motion.

## Materials and Methods

### Experimental Methods

#### Subjects

Ten healthy young subjects (6 males, 4 females; weight 73±14 kg and height 174.2±7.0 cm), aged 21–35 yr, were participated in the study. They all reported no musculoskeletal injuries or neurological disorders that might affect their ability to maintain balance. The procedures used in the experiment were approved by the Institutional Review Board at the University of Maryland and all the subjects gave written informed consent to participate as approved by the committee.

#### Experiment setup

**Figure 1:**
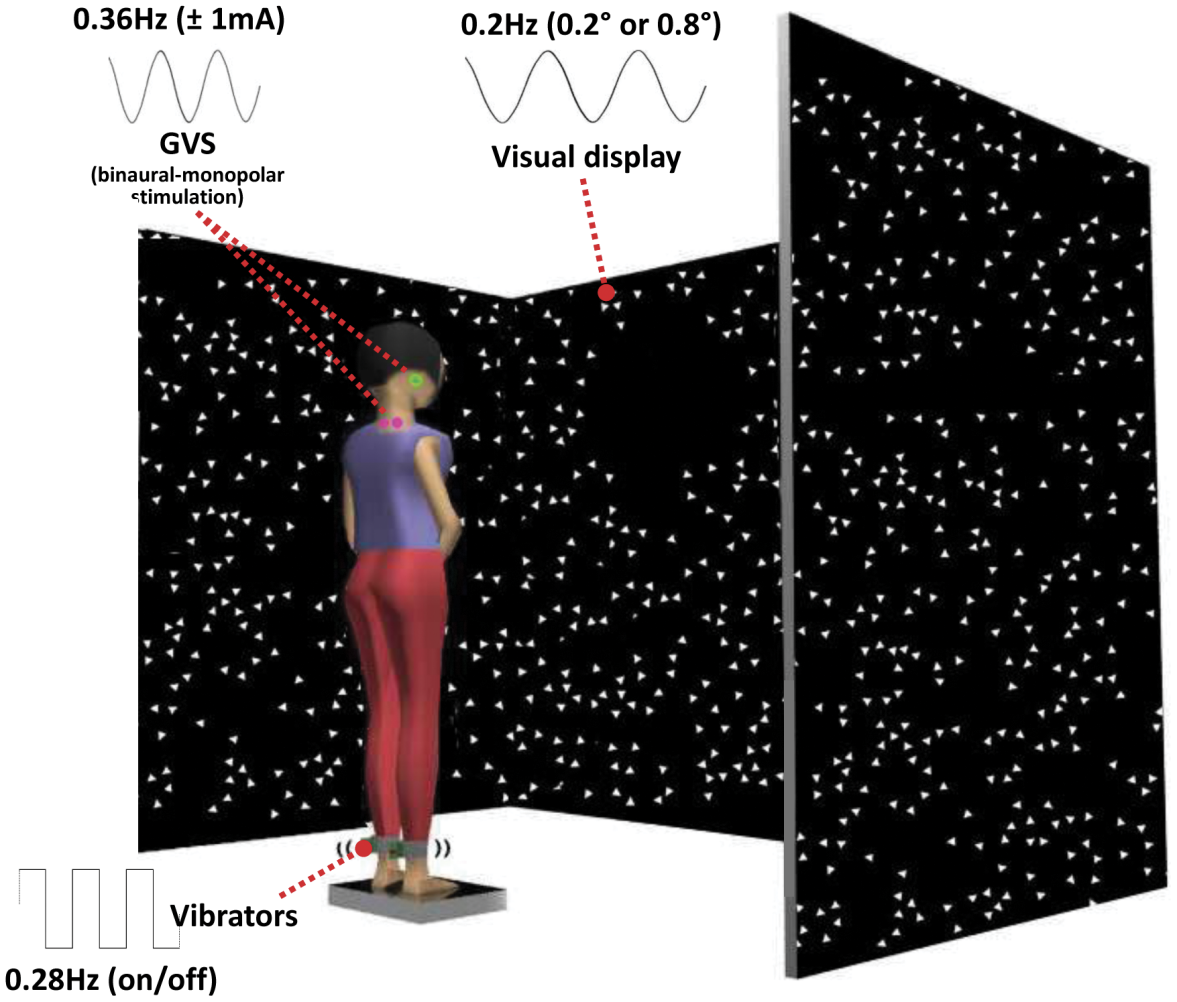
Experimental setup showing standing subject with simultaneous visual, vibration and galvanic vestibular perturbations. The visual stimulus at different amplitudes (0.2, 0.8 deg rotation about ankle axis) at 0.2 Hz, the 80 Hz vibratory stimulus to subject's bilateral Achilles tendons (stimulus turns on-off at 0.28 Hz) and a ±1 mA bilateral monopolar galvanic vestibular stimulus at 0.36 Hz were simultaneously applied.

Subjects stood in the middle of the visual cave and faced the front wall, as shown in Figure 1. Subjects assumed a foot position with heels at a distance of ∼11% of their heights and an angle of 14° between each foot and the midline [24]. The instruction to the subjects was to look straight ahead at the front screen and stand upright comfortably. Foot position was marked in order to be consistent throughout the experiment. Subjects were tested in four conditions; standing with low amplitude visual scene movement - vibration - GVS (L-V-G); standing with low amplitude visual scene movement - GVS – no vibration (L-G); standing with high amplitude visual scene movement - vibration - GVS (H-V-G); standing with high amplitude visual scene movement - GVS - no vibration (H-G). Four trials from each condition were run in randomized order, repeated seven times for a total of twenty eight trials. The length of each trial was 125 seconds with 5 seconds added at the beginning and end of each trial (total 135 seconds) to allow the visual and vestibular sensory perturbations to ramp up and ramp down. The ramping was not applied to the vibration signal because of the electronic functional limitation of the vibrator to turn on and off quickly to approximate a square-wave periodic stimulus.

#### Visual, proprioceptive and vestibular sensory perturbations

**Figure 2:**
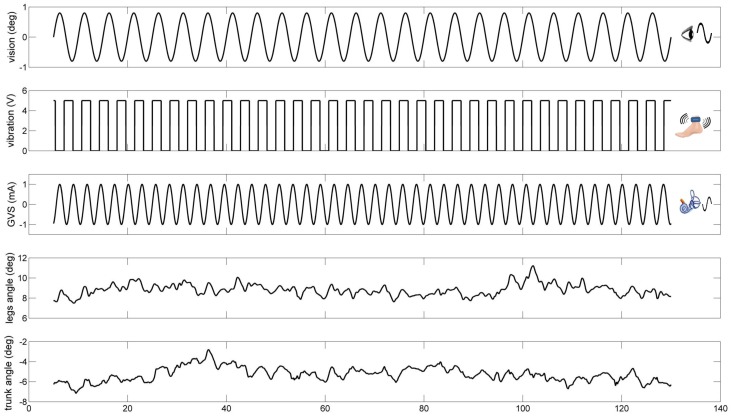
An example of a trial showing the stimulus signals and segments angles used for signal processing.

For the visual sensory perturbation, we used the visual display (called as visual cave) which was projected by JVC projectors (Model: DLA-M15U, Victor Company, Japan) to three mirrors, which reflected and rear-projected onto a visual cave consisting of three 2.67×3.33 m screens (Fakespace, Inc, Marshalltown, Iowa, USA). The visual display consisted of 500 randomly distributed white triangles (3.4×3.4×3 cm) on a black background. To reduce aliasing effects in the foveal region, no triangles were displayed within a horizontal band of ±5 degree at eye height. The frame rate of the visual display was 60 Hz. A visual signal was displayed as a visual rotation around ankle joint in anterior-posterior direction (i.e., sagittal plane). The visual stimulus was presented at different amplitudes (0.2 deg and 0.8 deg rotation about ankle axis) at 0.2 Hz to measure: the change in gain (weighting) to vision, an intramodal effect; and a change in gain to vibration and galvanic stimulation, both intermodal effects. For the proprioceptive sensory perturbation, bilateral vibration of Achilles tendons was applied through two 20 mm vibrator motors, driven at 80 Hz and 1 mm amplitude displacement. While vibration is a common technique, it is typically used in an always-on or always-off manner (e.g., Capicikova et al, 2006) [25]. For this study, we designed the vibrator to turn on and off quickly to approximate a square-wave periodic stimulus of a specified frequency with equal on and off time durations. The vibrators are enclosed in a hollow rectangular PVC container (3.5×3.8×3.5 cm) with a flexible recessed surface mounted on the contact face for comfortable fitting around the Achilles tendon. The enclosure was held in place by an elastic strap. The proprioceptive sensory perturbation was applied to different conditions (standing with vibration or standing without vibration) at 0.28 Hz. For the vestibular sensory perturbation, two linear isolated stimulators (Biopac Systems, Inc., Goleta, California, USA) were used as a binaural-monopolar galvanic vestibular stimulation (GVS). Independent stimuli were delivered to each side via a pair of circular electrodes secured over the mastoid process with an elastic headband and 2 cm ipsilateral to the T2 spinous process [26]. The electrodes were secured using adhesive tape and conductive electrode gel was applied at the electrode–skin interface to improve conductance. GVS consisted of a ±1 mA sinusoidal galvanic stimulus at 0.36 Hz and the polarity of stimulation was always the same for the two sides (binaural-monopolar GVS) to perturb subjects in the sagittal plane. GVS was applied to subjects in every trial of all conditions. Different frequencies for each stimulus were chosen so that we could measure a response to each modality independently and so that they did not share common low-order harmonics. Figure 2 shows an example of a trial showing the stimulus signals and segments angles used for signal processing.

#### Kinematics

**Figure 3:**
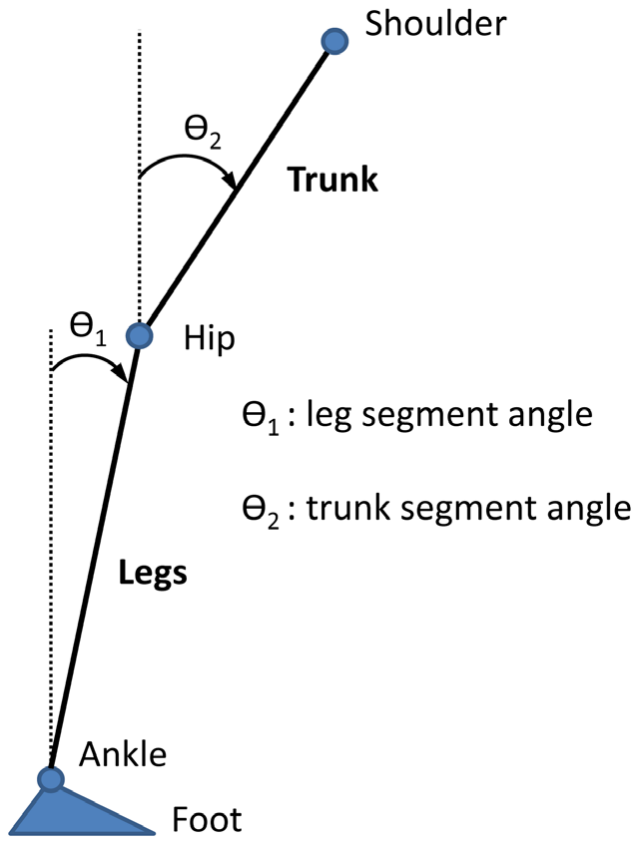
Schematic diagram of the body showing the leg segment angle *θ_1_* and trunk segment angle *θ_2_*.

Kinematics were captured by Vicon MX digital optical motion capture system with six infrared cameras (Vicon, UK). The head (the temple), shoulder (the scapula), hip (the greater trochanter), knee (the lateral femoral condyle), ankle (the lateral malleolus) and foot (the first metatarsal head) were measured by attaching twelve reflective markers on both sides of the subject to measure subject's anterior-posterior movement in the sagittal plane. The leg segment angle *θ_1_*(*t*) and trunk segment angle *θ_2_*(*t*) with respect to vertical were determined by the anterior-posterior (AP) and vertical displacement of the shoulder, hip and ankle markers, as shown in Figure 3. Kinematics were sampled at 120 Hz.

### Analysis

#### Spectral analysis

For any two signals *x*(*t*) and *y*(*t*), the power spectral densities (PSDs) *p_xx_*(*f*) and *p_yy_*(*f*) and cross spectral density(CSD) *p_xy_*(*f*), where *f* is frequency, were computed using Welch's method [27] with 50-s Hanning windows and 50% overlap and then averaged across trials. Note that 25 s is an integer multiple of the periods of all three perturbation signals, so the 50-s window contains an integer number of cycles of each perturbation signal. The frequency response function (FRF) is the CSD divided by the PSD of the input. Gain, the absolute value of the FRF, is the amplitude of the output divided by the amplitude of the input at each driving frequency. For example, if the amplitudes of a segment angle response and the visual perturbation at the driving frequency are the same, the unitless gain (deg/deg) will equal one. Phase of the FRF is a measure of the temporal relationship between the input and output; the output may lead the input (positive values) or lag behind it (negative values). Bootstrap standard errors were also computed for gains and phases using 1000 bootstrap resamples [28].

#### Statistical analysis

In order to determine effects due to a change in visual amplitude and effect due to vibration on/off, values for gain and phase of leg angle θ_1_(t) and trunk angle θ_2_(t) relative to visual stimulus signal and GVS were respectively assessed using visual amplitude effect (low vs high amplitude visual scene movement)×vibration effect (vibration or no vibration) two way repeated-measures ANOVA. For the vibration perturbation, the cluster of FRF values for different subjects surrounded the origin in the complex plane. Therefore, to test gain and phase we analyzed the FRF values using the maximum-likelihood method of Jeka et al. (2008) [29], which is designed for this case. For the visual and GVS perturbations, FRF values were clustered away from the origin, so we used the simpler method of computing gain and phase for each subject and then analyzing gain and phase separately using ANOVAs. Therefore, the maximum-likelihood method was applied to determine differences of gain and phase of leg and trunk angle relative to vibration between L-V-G and H-V-G. A gain/phase response can be measured only with vibration turned on (L-V-G and H-V-G) because it cannot be measured with vibration turned off (L-G and H-G). Differences were accepted to be significant at p<0.05.

## Results

**Figure 4:**
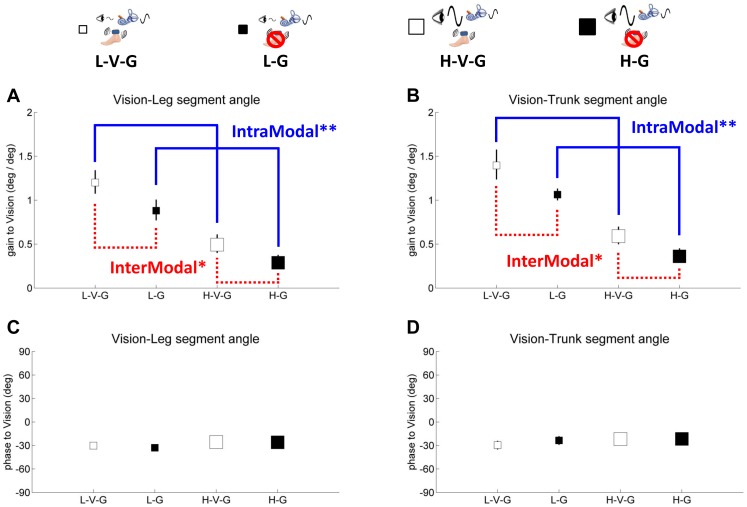
Gain and phase of segment angles relative to vision, showing intramodal visual reweighting (blue solid line) and intermodal visual-proprioceptive reweighting (red dashed line). Blue color indicates gain responses about changing of visual amplitude and red color indicates gain responses about vibration. Solid line indicates intramodal reweighting and dashed line indicates intermodal reweighting. The asterisk indicates significant reweighting effects (** for p<.01 and * for p<.05). **A,** gain of the leg segment angle relative to vision. **B,** gain of the trunk segment angle relative to vision. **C,** phase of the leg segment angle relative to vision. **D,** phase of the trunk segment angle relative to vision. Error bars denote bootstrap standard errors.

**Figure 5:**
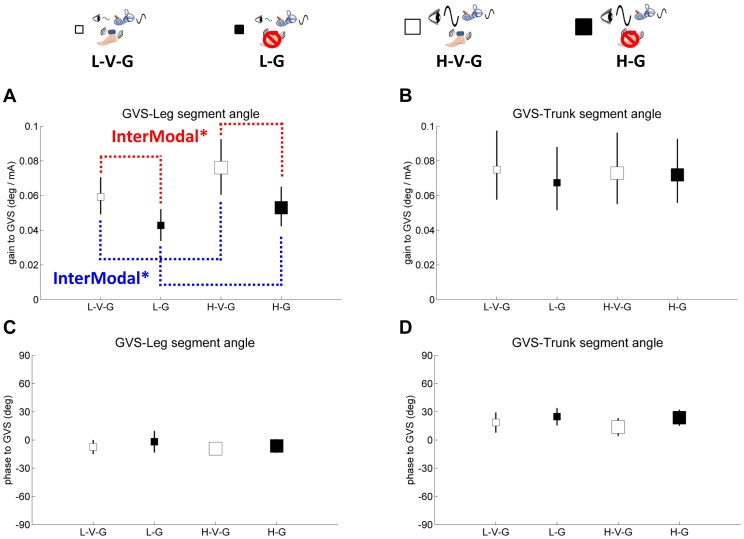
Gain and phase of segment angles relative to galvanic vestibular stimulation (GVS), showing intermodal reweighting. Blue color indicates gain responses about changing of visual amplitude and red color indicates gain responses about vibration. Dashed line indicates intermodal reweighting. The asterisk indicates significant reweighting effects (* for p<.05). **A,** gain of the leg segment angle relative to GVS. **B,** gain of the trunk segment angle relative to GVS. **C,** phase of the leg segment angle relative to GVS. **D,** phase of the trunk segment angle relative to GVS. Error bars denote bootstrap standard errors.

**Figure 6:**
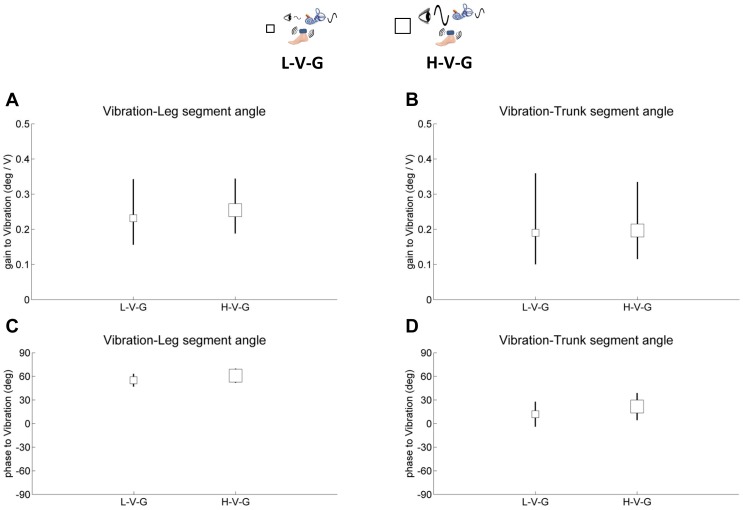
Gain and phase of segment angles relative to vibration. **A,** gain of the leg segment angle relative to vibration. **B,** gain of the trunk segment angle relative to vibration. **C,** phase of the leg segment angle relative to vibration. **D,** phase of the trunk segment angle relative to vibration. Error bars denote bootstrap standard errors.

Leg and trunk gain and phase responses are shown on separate plots relative to each sensory perturbation in Figures 4–6. Small white square represents standing with low amplitude visual scene movement – vibration – GVS (L-V-G), small black square represents standing with low amplitude visual scene movement - GVS – no vibration (L-G), large white square represents standing with high amplitude visual scene movement - vibration - GVS (H-V-G) and large black square represents standing with high amplitude visual scene movement - GVS - no vibration (H-G). It is most informative to digest how each sensory modality reacts to the same condition by comparing gains and phases across Figures 4–6.

### Gain responses to visual amplitude change

In Figure 4A, leg and trunk gain relative to vision decreases from the L-V-G condition to the H-V-G condition and from the L-G condition to the H-G condition (F_(1,9)_ = 106.0, p<.001), reflecting a clear *intramodal* downweighting of vision as visual scene amplitude increased, consistent with previous studies [16], [30], [31]. This effect was observed for trunk gain relative to vision as well (F_(1,9)_ = 91.4, p<.001), as shown in Figure 4B. We also observed *intermodal* effects due to a change in visual amplitude. For example, Figure 5A illustrates a significant increase in leg gain relative to the GVS stimulus (F_(1,9)_ = 5.9, p<.04) when visual amplitude increases (L-V-G to H-V-G, L-G to H-G), reflecting an intermodal vestibular upweighting to compensate for visual downweighting. These effects were not observed for trunk gain relative to the GVS stimulus. There were also not any significant intermodal effects of visual amplitude change on leg/trunk gain relative to vibration (Figure 6).

### Gain responses to vibration on/off

Intramodal effects were not observable with vibration because a gain response cannot be measured with vibration turned off. However, the effect of turning vibration on/off was clearly observed through intermodal effects on visual and GVS stimulation. When vibration is turned off in the L-G and H-G conditions, leg gain relative to visual (Figure 4A) and GVS (Figure 5A) stimuli decrease from the L-V-G and H-V-G conditions, respectively (F_(1,9)_ = 5.7, p<.05; F_(1,9)_ = 5.4, p<.05). These intermodal effects suggest that vibration changes processing of proprioceptive information at the foot/ankle, forcing the nervous system to compensate by upweighting vision and vestibular information in the L-V-G and H-V-G conditions. Similar effects were observed for trunk gain for the visual stimulus in Figure 4B (F_(1,9)_ = 5.1, p<.05), but no significant effects were observed for trunk gain relative to the GVS stimulus in Figure 5B.

### Phase

Phase of the leg/trunk segments relative to each of the sensory stimuli shown in Figures 4–6 C–D indicated no differences across conditions. However, absolute differences in phase were observed relative to the mode of sensory stimulation. Leg/trunk segment angles displayed similar phase lags of 25 deg∼30 deg relative to vision and phase advances of 50 deg and 25 deg, respectively, relative to vibration. Relative to the GVS stimulus, leg phase was zero and trunk phase was advanced by ∼25 deg.

## Discussion

In this study, we investigated how the primary trio of modalities (visual, vestibular and proprioceptive system) that are crucial for flexible postural control are processed in order to provide estimates of self-motion that are optimal to stabilize upright stance. Theoretically, one can decompose sensory feedback into individual feedback components from each sensory modality. However, these modalities are not processed independently; when sensory conditions change an adaptive process known as sensory re-weighting changes the relative importance of each modality in a coordinated fashion. For the first time, we experimentally elucidate the sensor fusion process for all three modalities simultaneously during standing to understand how the nervous system adjusts the emphasis on each modality under different combinations of static and dynamic sensory input.

In order to achieve these results, we used vibration in a novel way to induce and measure the effects of sensory re-weighting. Vibration is a common technique to perturb proprioceptive input; it can either be applied in a sustained manner [25], [32] or turned on and off stochastically [33], [34]. The second approach allows one to measure responses across a range of frequencies. Here we turned vibration on and off periodically to focus on a single stimulus frequency, allowing us to detect the effects of sensory re-weighting more precisely at this frequency. We designed the vibrator to turn on and off quickly to approximate a square-wave periodic stimulus at the specified frequency, allowing calculation of gain/phase of trunk and leg segments relative to the vibration stimulus. This was critical in allowing us to compare changes in gain/phase between each modality and allow interpretation of how each was reweighted as visual amplitude was manipulated.

### Gain

The results illustrate both intramodal and intermodal reweighting of trunk/leg segment responses relative to the three sensory perturbations. When visual amplitude was increased from the L-V-G to the H-V-G condition, leg/trunk segment gain relative to vision decreases, suggesting downweighting of vision, consistent with previous studies [16], [30], [31]. With the increase in visual amplitude, we also observed an increase in vestibular gain relative to the galvanic stimulus, suggesting an intermodal upweighting of the vestibular information to compensate for visual downweighting. Intermodal upweighting of the proprioceptive channel was not observed. This may be due to the “local” nature of the vibratory stimulus. While the visual and vestibular modalities are anatomically organized so that a stimulus can bias the entire peripheral organ, muscle spindles are distributed throughout the musculature. A vibration localized to the Achilles tendon biases the sensory signals from an important muscle group related to posture, but there are many other muscles that play an active role in maintaining upright stance. Thus, intermodal effects of visual change on proprioception may be difficult to achieve because the bias is distributed across many proprioceptive sensors and in different body segments.

Interestingly, intermodal effects of vibration on visual and vestibular gain were observed. The increase in trunk/leg gain relative to the visual and GVS stimuli from the L-G and H-G conditions to the L-V-G and H-V-G conditions, respectively, shows how the visual and vestibular modalities are upweighted when vibration is turned on. These intermodal effects are observed because vibration changes proprioceptive inputs at the ankles thereby making these inputs a less reliable indicator of self-motion. Disruption of proprioception, even locally at the ankles, seems to affect visual and vestibular processing much more than a change in the visual stimulus affects proprioception. This intermodal asymmetry emphasizes the delicate interplay between the modalities. This interplay of intramodal (vision) and intermodal (proprioceptive and vestibular) effects reflect a central “sensor fusion” process that continuously incorporates sensory input to generate the most reliable estimates of self-motion for the maintenance of upright equilibrium.

We also observed effects based upon body segment, as the trunk and leg segments did not respond equivalently to different types of stimuli. The contrast between segment effects based upon visual and vestibular gain are particularly striking. Intramodal and intermodal effects on visual gain were almost equivalent across the leg and trunk segments. This is consistent with the view that although the nervous system interprets visual information in the context of multi-segment dynamics, responses to visual information involves a single control signal that determines the activation of all muscles [35], [36]. In contrast, intermodal effects of vision or vibration on vestibular gain were observed for the leg segment, with no significant effect on the trunk segment. This suggests that vestibular information not only provides information about intersegmental control, but that vestibular information is also used to differentially activate different muscles, consistent with previous work [37]. Clearly each modality is playing a specific role in controlling the multi-segment body.

### Phase

We did not observe differences in trunk/leg phase relative to each stimulus as a function of condition. However, phase differences across modality were observed, which may be due either to the processing properties of the modality or the different frequencies at which each was presented. Our technique to investigate “sensor fusion”stems from linear systems theory. Subjects are typically “driven” by an oscillating pattern of sensory information. The resulting postural responses of the body are measured to determine system properties [38]. For example, the sinusoidal vertical axis rotation (SVAR) technique rotates seated subjects at a range of frequencies to measure the gain and phase of eye movements in the dark as a measure of vestibular function [39], [40]. Likewise, an oscillating visual “moving room” has been used to demonstrate the coupling of visual information with whole-body posture [2], [41]–[43]. Such methods have consistently found that as the frequency of stimulation increases, the phase of the response decreases, due to factors such as time delays and the low-pass filtering properties of musculotendon and body dynamics. Note that our results are inconsistent with these factors being equivalent for all modalities, as the modality presented at the lowest frequency (vision at 0.2 Hz) had the largest phase lag (≈−30 deg) while modalities presented at higher frequencies (vibration at 0.28 Hz, GVS at 0.36 Hz) displayed either zero phase lag or a phase lead, respectively. This is consistent with known differences among time delays for different modalities. Proprioceptive information has the shortest time delays, with monosynaptic pathways that can process information as quickly as 40–50 ms [44]. Vision, at the opposite extreme, is relatively slow, with time delays as long as 150–200 ms [45], [46]. Vestibular processing delays are thought to be somewhere between these extremes [47].

### Vestibular input – sensor fusion

Vestibular inputs provide absolute information about the body's orientation, whereas visual and proprioceptive inputs provide relative information (relative to visual scene and relative to the support surface) about the body's orientation. Since in everyday life the visual scene and/or support surface can move, this means that visual and proprioceptive inputs do not necessarily provide veridical information about the body's orientation. Therefore, vestibular input has been considered to provide the sole source of veridical information about self-motion, serving as a reference against which other sensory inputs are evaluated when conflicts among inputs from multiple senses occur. For example, DeAngelis and Angelaki (2012) suggest that vestibular signals could be of specific importance in dealing with object motion because the vestibular modality provides an independent source of information about head movements that may help to identify optic flow that is inconsistent with self-motion induced by moving objects [48]. Similarly, Mahboobin et al. (2009) model sensory reweighting of graviceptive/vestibular and proprioceptive inputs for the control of upright stance under the assumption that graviceptive inputs provide veridical but noisy orientation information [49]. When a large conflict occurs between the graviceptive and proprioceptive inputs, the graviceptive input is “trusted” and its weight is increased while the proprioceptive weight is decreased.

Our observed changes in gain to the GVS perturbation were consistent with the Mahboobin et al. model, assuming that the model is extended to include a visual input that, like the proprioceptive input, is non-veridical. Vibration of the Achilles tendon increased the GVS gain, consistent with an up-weighting of vestibular information due to a conflict between vestibular and proprioceptive inputs. Similarly, GVS gain increased with increasing amplitude of visual-scene motion. However, these intermodal effects are also consistent the model of Carver et al. (2005; 2006) [50], [51], which reweights two sensory inputs without assuming that either one is veridical. Instead, it changes sensory weights in whichever direction improves postural control performance, as measured by mean-squared specified ankle torque. As an avenue for future research, we can distinguish between these two models by changing the GVS amplitude. With increasing GVS amplitude, the Mahboobin et al. model would increase the GVS gain, since the model would falsely attribute the increased sensory conflicts to increased errors in non-vestibular sensory modalities. In contrast, the Carver et al. model would decrease the GVS gain, since doing so would increase postural control performance.

## Conclusions

Simultaneous manipulation of the three sensory modalities that are critical for upright stance control demonstrate their interplay for stable and flexible control of upright stance. This is the first time, to our knowledge, that the interplay between the three primary modalities for postural control has been clearly delineated, illustrating a central process that fuses these modalities for accurate estimates of self-motion.

## References

[1] Horak FB, Macpherson JM (1996) Postural orientation and equilibrium. Handbook of Physiology, Exercise: Regulation and Integration of Multiple Systems. New York: Oxford, sect 12: 255–292.

[2] Berthoz A, Lacour M, Soechting JF, Vidal PP (1979) The role of vision in the control of posture during linear motion. Progress in Brain Research 50: 197–209. 551426 10.1016/S0079-6123(08)60820-1

[3] Bronstein AM (1986) Suppression of visually evoked postural responses. Experimental Brain Research 63: 655–658. 3489640 10.1007/BF00237488

[4] Dijkstra TM, Schöner G, Gielen CC (1994) Temporal stability of the action-perception cycle for postural control in a moving visual environment. Experimental Brain Research 97: 477–486. 8187859 10.1007/BF00241542

[5] Lee DN, Lishman JR (1975) Visual proprioceptive control of stance. Journal of Human Movement Studies 1: 87–95.

[6] Lestienne F, Schoechting JF, Berthoz A (1977) Postural readjustment induced by linear motion of visual scenes. Experimental Brain Research 28: 363–384. 885185 10.1007/BF00235717

[7] van Asten WN, Gielen CC, Diener van der Gon JJ (1988) Postural adjustments induced by simulated motion of differently structured environments. Experimental Brain Research 73: 371–383. 3215313 10.1007/BF00248230

[8] Allum JH (1983) Organization of stabilizing reflex responses in tibialis anterior muscles following ankle flexion perturbations of standing man. Brain Res 264: 297–301. 6850299 10.1016/0006-8993(83)90828-4

[9] Jeka JJ, Schöner G, Dijkstra T, Ribeiro P, Lackner JR (1997) Coupling of fingertip somatosensory information to head and body sway. Experimental Brain Research 113: 475–483. 9108214 10.1007/pl00005600

[10] Johansson R, Magnusson M, Akesson M (1988) Identification of Human Postural Dynamics. IEEE Trans Biomed Eng 35: 858–869. 3192235 10.1109/10.7293

[11] Kavounoudias A, Gilhodes JC, Roll R, Roll JP (1999) From balance regulation to body orientation: two goals for muscle proprioceptive information processing, xperimental. Brain Research 124: 80–88. 10.1007/s002210050602 9928792

[12] Day BL, Severac Cauquil A, Bartolomei L, Pastor MA, Lyon IN (1997) Human body-segment tilts induced by galvanic stimulation: a vestibularly driven balance protection mechanism. J physiol 500: 661–672. 9161984 10.1113/jphysiol.1997.sp022051 PMC1159417

[13] Hlavacka F, Njiokiktjien C (1985) Postural responses evoked by sinusoidal galvanic stimulation of the labyrinth. Influence of head position. Acta Otolaryngol 99: 107–112. 3872005 10.3109/00016488509119152

[14] Johansson R, Magnusson M, Fransson PA (1995) Galvanic vestibular stimulation for analysis of postural adaptation and stability. IEEE Trans Biomed Eng 42: 282–292. 7698784 10.1109/10.364515

[15] Nashner LM, Wolfson P (1974) Influence of head position and proprioceptive cues on short latency postural reflexes evoked by galvanic stimulation of the human labyrinth. Brain Res 67: 255–268. 4470421 10.1016/0006-8993(74)90276-5

[16] Peterka RJ (2002) Sensorimotor integration in human postural control. J Neurophysiol. Sep 88 3: 1097–1118. 10.1152/jn.2002.88.3.1097 12205132

[17] Peterka RJ, Loughlin PJ (2004) Dynamic regulation of sensorimotor integration in human postural control. Journal of Neurophysiology 91: 410–423. 13679407 10.1152/jn.00516.2003

[18] van der Kooij H, Jacobs R, Koopman B, van der Helm F (2001) An adaptive model of sensory integration in a dynamic environment applied to human stance control. Biol Cybern 84 2: 103–115. 11205347 10.1007/s004220000196

[19] Mahboobin A, Beck C, Moeinzedah M, Loughlin PJ (2002) Analysis and validation of a human postural control model. Proceeding of the American Control Conference 4122–4128.

[20] Kiemel T, Oie KS, Jeka JJ (2002) Multisensory fusion and the stochastic structure of postural sway. Biological Cybernetics 87: 262–277. 12386742 10.1007/s00422-002-0333-2

[21] Oie KS, Kiemel T, Jeka JJ (2001) Human multisensory fusion of vision and touch: detecting nonlinearity with small changes in the sensory environment. Neuroscience Letters 315: 113–116. 11716976 10.1016/s0304-3940(01)02348-5

[22] Mahboobin A, Loughlin PJ, Redfern MS, Sparto PJ (2005) Sensory re-weighting in human postural control during moving-scene perturbations. Experimental Brain Research 167: 260–267. 16025292 10.1007/s00221-005-0053-7

[23] Mahboobin A (2008) Computational and Robotic Models of Human Postural Control. Doctoral Dissertation, University of Pittsburgh.

[24] McIlroy WE, Maki BE (1997) Preferred Placement of the Feet During Quiet Stance: Development of a Standardized Foot Placement for Balance Testing. Clinical Biomechanics 12: 66–70. 11415674 10.1016/s0268-0033(96)00040-x

[25] Capicikova N, Rocchi I, Hlavacka F, Chiari I, Cappello A (2006) Human postural response to lower leg muscle vibration of different duration. Physiol Res 55 1: S129–S134. 17177621 10.33549/physiolres.930000.55.S1.129

[26] Day BL, Marsden JF, Ramsay E, Mian OS, Fitzpatrick RC (2010) Non-linear vector summation of left and right vestibular signals for human balance. J physiol 508 4: 671–682. 10.1113/jphysiol.2009.181768 PMC2828139 20026614

[27] Bendat JS, Piersol AG (2000) Random data: Analysis and measurement procedures, 3rd edition. New York: Wiley.

[28] Zoubir AM, Boashash B (1998) The bootstrap and its application in signal processing. IEEE Signal Processing Magazine 15: 56–76.

[29] Jeka JJ, Oie KS, Kiemel T (2008) Asymmetric adaptation with functional advantage in human sensorimotor control. Exp Bran Res 191: 453–463. 10.1007/s00221-008-1539-x PMC2731555 18719898

[30] Kiemel T, Oie KS, Jeka JJ (2006) Slow Dynamics of Postural Sway Are in the Feedback Loop. J Neurophysiol 95: 1410–1418. 16192341 10.1152/jn.01144.2004 PMC2717851

[31] Oie KS, Kiemel T, Jeka JJ (2002) Multisensory fusion: Simultaneous re-weighting of vision and touch for the control of human posture. Cognitive Brain Research 14: 164–176. 12063140 10.1016/s0926-6410(02)00071-x

[32] Lackner JR, Rabin E, Dizio P (2000) Fingertip contact suppresses the destabilizing influence of leg muscle vibration. J Neurophysiol 84: 2217–2224. 11067967 10.1152/jn.2000.84.5.2217

[33] Johansson R, Magnusson M, Fransson PA, Karlberg M (2001) Multi-stimulus multi-response posturography. Mathematical Biosciences 174: 41–59. 11595256 10.1016/s0025-5564(01)00075-x

[34] Fransson PA, Hjerpe M, Johansson R (2007) Adaptation of multi-segmented body movements during vibratory proprioceptive and galvanic vestibular stimulation. Journal of Vestibular Research 17: 47–62. 18219104

[35] Kiemel T, Elahi AJ, Jeka JJ (2008) Identification of the Plant for Upright Stance in Humans: Multiple Movement Patterns From a Single Neural Strategy. J Neurophysiol 100: 3394–3406. 18829854 10.1152/jn.01272.2007 PMC2604857

[36] Kiemel T, Zhang Y, Jeka JJ (2011) Visual flow is interpreted relative to multisegment postural control. J of Motor Behavior 43 3: 237–246. 10.1080/00222895.2011.568991 21534025

[37] Creath R, Kiemel T, Horak FB, Jeka J (2008) The role of vestibular and somatosensory systems in intersegmental control of upright stance. J of Vestibular Research 18: 39–49. PMC2938746 18776597

[38] Jeka JJ, Oie KS, Schoner G, Dijkstra T, Henson E (1998) Position and Velocity Coupling of Postural Sway to Somatosensory Drive. Journal of Neurophysiology 79: 1661–1674. 9535937 10.1152/jn.1998.79.4.1661

[39] Krebs DE, Gill-body KE, Riley PO, Parker SW (1993) Double-blind, placebo-controlled trial of rehabilitation for bilateral vestibular hypofunction: preliminary report. Otalaryngol. Head Neck Surg 109: 735–741. 10.1177/019459989310900417 8233513

[40] Howard IP (1982) Human Visual Orientation. New York: Wiley.

[41] Dijkstra TM, Schöner G, Giese MA, Gielen CCAM (1994) Frequency dependence of the action-perception cycle for postural control in a moving visual environment: Relative phase dynamics. Biological Cybernetics 71: 489–501. 7999875 10.1007/BF00198467

[42] Peterka RJ, Benolken MS (1995) Role of somatosensory and vestibular cues in attenuating visually induced human postural sway. Experimental Brain Research 105: 101–110. 7589307 10.1007/BF00242186

[43] Soechting J, Berthoz A (1979) Dynamic role of vision in the control of posture in man. Experimental Brain Research 36: 551–561. 477782 10.1007/BF00238522

[44] Campbell SA (2007) Time delays in neural systems. In: McIntosh AR and Jirsa VK, editors. Handbook of brain connectivity. New York, NY: Springer Verlag.

[45] Gabor S (2009) Delay effects in the human sensory system during balancing. Phil Trans R Soc 367: 1195–1212. 10.1098/rsta.2008.0278 19218159

[46] Nijhawan R (2008) Visual prediction: Psychophysics and neurophysiology of compensation for time delays. Behavioral and Brain Sciences 31: 179–239. 18479557 10.1017/S0140525X08003804

[47] Fitzpatrick R, McCloskey DI (1994) Proprioceptive, visual and vestibular thresholds for the perception of sway during standing in humans. Journal of Physiology 478 1: 173–186. 7965833 10.1113/jphysiol.1994.sp020240 PMC1155655

[48] DeAngelis GC, Angelaki DE (2012) Visual-Vestibular Integration for Self-Motion Perception. In: Murray MM, Wallace Mt, editors. The Neural Bases of Multisensory Processes, Boca Raton(FL): CRC Press; 2012. Chapter 31.

[49] Mahboobin A, Loughlin P, Atkeson C, Redfern M (2009) A mechanism for sensory re-weighting in postural control. Med Biol Eng Comput 47: 921–929. 19326162 10.1007/s11517-009-0477-5 PMC4899834

[50] Carver S, Kiemel T, van der Kooij H, Jeka JJ (2005) Comparing internal models of the dynamics of the visual environment. Biol Cybern 92: 147–163. 15703940 10.1007/s00422-004-0535-x

[51] Carver S, Kiemel T, Jeka JJ (2006) Modeling the dynamics of sensory reweighting. Biol Cyvern 95: 123–134. 10.1007/s00422-006-0069-5 16639582

